# Effect of buttermilk and skimmed milk powder on the properties of low-fat yoghurt

**DOI:** 10.1007/s13197-021-05227-w

**Published:** 2021-08-12

**Authors:** Anna Garczewska-Murzyn, Michał Smoczyński, Natalia Kotowska, Katarzyna Kiełczewska

**Affiliations:** Department of Dairy Science and Quality Management, Faculty of Food Sciences, University of Warmia and Mazury in Olsztyn, Oczapowskiego Str. 7, 10-719, Olsztyn, Poland

**Keywords:** Yoghurt, Buttermilk powder, Phospholipids, Acidity, Syneresis

## Abstract

**Abstract:**

The aim of the study was to determine the potential of using buttermilk and skimmed milk powders as additives to standardize the dry matter content of milk in the production of low-fat yoghurt. A batch of yoghurt was produced using a starter culture of *Lactobacillus delbruecki ssp. bulgaricus* and *Streptococcus thermophilus*. The rates of milk acidification and pH levels were similar for both variants of yoghurt. After chilled storage (21 days), the yoghurt produced from milk supplemented with buttermilk powder was found to contain higher (*P* ≤ 0.05) levels of lactic acid (1.179%) than that supplemented with skimmed milk (1.154%). The use of buttermilk powder allowed reducing (not significantly, *P* > 0.05) syneresis in the stored yoghurt. The milk fat in the buttermilk–supplemented yoghurt showed lower (*P* ≤ 0.05) phospholipids content and exhibited slightly higher phospholipids loss during storage than the yoghurt produced from milk with addition of milk powder. No differences were found between the profile of fatty acids between the yoghurts enriched with skimmed milk powder and those enriched with buttermilk powder. Buttermilk can be used as an additive to produce a novel yoghurt type with modified functional features.

**Research Highlights:**

The use of buttermilk powder did not affect fermentation process, however increased lactic acid content and water-holding capacity of yoghurt.

The yoghurts with added buttermilk contained less phospholipids when compared with yoghurts supplemented with milk powder.

Buttermilk powder can be incorporated as an ingredient in production of novel yoghurt type with improved functional features.

## Introduction

Both milk and dairy products are food items of high nutritional and dietary value due to their biologically active ingredients (Korhonen 2009). Particularly noteworthy are powdered milk products—powdered milk, powdered buttermilk, powdered whey, concentrates of all milk proteins and whey protein concentrate—which can serve as interesting food additives due to their useful properties and nutritional values, mainly due to their proteins (Silva and O'Mahony 2017). Buttermilk as a powdered product can potentially boost the nutritional and health-promoting properties of foodstuffs, as well as modify their structure and consistency. Buttermilk contains all of the water-soluble milk constituents, including milk proteins, lactose and minerals, as well as large quantities of milk fat globule membranes (MFGM) (Smoczyński et al. 2012). During the churning process, fragments of the membranes are released into the milk plasma, resulting in higher levels of polar lipids in the dry matter of buttermilk (Dewettinck et al. 2008; Vanderghem et al. 2010) and in the milk fat (Contarini and Povolo 2013; Rombaut and Dewettinck 2006) when compared with whole milk, cream or butter. It can serve as valuable raw material for extracting MFGM components, whose functional and health-promoting properties have been noted by many researchers. The components of the membrane, including polar lipids and MFGM proteins, support the healthy functioning of the body, and may thus be added to food products to enrich their composition and improve their nutritional and biological values (Anto et al. 2020; Conway et al. 2014; Contarini and Povolo 2013; Corredig and Dalgleish 1997; Dewettinck et al. 2008; Fontecha et al. 2020; Spitsberg 2005; Vanderghem et al. 2010). Given their functional features, i.e. their structure-forming, emulsifying and water-binding properties, buttermilk and MFGMs can be utilized in cheesemaking, yoghurt-production, and other technological processes (Dewettinck et al. 2008; Vanderghem et al. 2010).

Yoghurt is a dairy product obtained through fermentation with starter cultures containing *Lactococcus delbruecki ssp. bulgaricus* and *Streptococcus thermophilus* bacteria (Horiuchi and Sasaki 2012). It belongs to the group of concentrated fermented milks. The concentration of yoghurt dry matter components is an important determinant of the texture, composition, and rheology of the final product. One of the methods used to increase the concentration of milk dry matter components is to add powdered milk or powdered milk proteins prior to the acidification (Jørgensen et al. 2019). Fortification of the dry matter content through the addition of sweet buttermilk prevents syneresis and improves the structure and texture of low-fat yoghurt (Trachoo and Mistry 1998). Milk fat, both in terms of its dispersion and fatty acid composition, plays an important role in developing the taste and consistency of yoghurt. For this reason, yoghurt substitutes that could be used in low-fat products are sought these days. Through the use of buttermilk, sensory attributes of low-fat yoghurts can be modified to resemble those of full-fat yoghurt (Zhao et al. 2018).

Due to the beneficial effects of buttermilk in fermented milk technology, the aim of the present study was to determine the potential of using buttermilk and skimmed milk powders as additives in the production of low-fat yoghurt.

## Materials and methods

### Research material

The research material encompassed bulk milk obtained during winter from farms in Warmia-Mazury Voivodeship, as well as sweet buttermilk powder and skimmed milk powder produced in an industrial facility.

### Study design

Raw milk was centrifuged at 45 °C in a skimming separator (Gea Westfalia Separator System GmbH, Oelde, Germany) to skim the milk and standardize its fat content. The dry matter content of milk was standardized to 13% using buttermilk powder (BMP) and skimmed milk powder (SMP). Then, the cream with 30% content of fat was added to the milk with buttermilk powder and milk with skimmed milk powder to standardize their fat content to 1.5%. The prepared milk samples were subjected to a two-step homogenization process at a temperature of 65 °C and pressures of p_1_ = 15 MPa, p_2_ = 3 MPa using a PandaPlus 2000 homogenizer (Gea Niro Soavi, Italy), as well as to a pasteurization process at 95 °C for 60 s using a Thermomix TM 31 (Vorwerk, Wuppertal, Germany). After the milk was chilled to 45 °C, a freeze-dried YC-X11 Yo-flex starter culture (Chr. Hansen, Hoersholm, Denmark) containing *Lactobacillus delbruecki ssp. bulgaricus* and *Streptococcus thermophilus* species was added as an inoculum. The samples were then thoroughly mixed, placed into plastic cups and incubated at 45 °C until pH of 4.6–4.7 was reached. After attaining target pH, the yoghurt was chilled to 4 °C and stored for 21 days. The tested samples included the milk with buttermilk powder (MBMP) and the milk with skimmed milk powder (MSMP) used for standardization, as well yoghurts produced from these sources: yoghurt made from milk with buttermilk powder (YBMP) and yoghurt made from milk with skimmed milk powder (YSMP), that were chill-stored for 1 day, 7 days and 21 days.

### Chemical composition

The content of total solids was determined according to AOAC guidelines (AOAC International, 2007; method 990.20; 33.2.44). Fat content was determined with the Gerber method according to standard IDF 238 (ISO 19662, 2018).

### Acidity

The pH value was measured using a Testo 205 pH-meter (Testo, Titisee-Neustadt, Germany). Milk (100 mL) was titrated with 0.25 M NaOH using phenolphthalein as the indicator. The results were expressed as Soxhlet-Henkel degrees (°SH). Lactic acid content were determined according to AOAC guidelines (AOAC International, 2007; method 947.05; 33.2.06).

### Syneresis

Syneresis was measured by centrifuge testing (Amatayakul et al. 2006). The yoghurt sample was stirred 20 times in the clockwise and counterclockwise direction with a thin spatula. Ca. 20 g of yoghurt were transferred into polypropylene centrifuge tubes and left at 4 °C for 2 h to stabilize. Afterward, the samples were spun in a Heraeus Megafuge 16 (Thermo Fisher, Waltham, USA) at 3000 rpm and 10 °C for 15 min. After centrifugation, the separated whey was weighed. The syneresis was expressed as the percentage of the separated whey relative to the weight of the centrifuged sample.

### Extraction of the lipid fraction

Fat was extracted from the yoghurt using the method described by Hara and Radin (1978). A 3:2 mixture of hexane and isopropanol was used for fat extraction. The samples were measured out into ground-glass conical flasks, after which 18 mL of a solvent mixture was added per 1 g of the test material. The samples were then vigorously stirred for 60 s. An aqueous solution of sodium sulphate (1 g of salt dissolved in 15 cm^3^ of water) was mixed at a 12:1 ratio. After thorough phase separation, the upper layer was collected into glass beakers and the solvent was evaporated at 30 °C in a Binder BF 53 incubator (Binder, Tuttlingen, Germany).

### Phospholipids content

The phospholipids content was established using the colorimetric method, after Stewart (1980). The extracted fat was weighed out into a polypropylene centrifuge tube and dissolved in 2 mL of chloroform. Next, 1 mL of a thiocyanate reagent (27 g of ferric chloride and 30 g of ammonium thiocyanate in 1 L of distilled water) was added to the tube. The mixture was spun in a Heraeus Megafuge 16 (Thermo Fisher, Waltham, USA) at 1500 rpm for 7 min. After removing the upper red layer, the absorbance of the lower layer was measured at 488 nm using a Helios Beta spectrophotometer (Spectronic Unicam, United Kingdom). The content of phospholipids in 1 mg of fat was established based on the curves of absorbance correlation with the phospholipids concentration. Standard solutions of phospholipids (Sigma-Aldrich, Merck, Darmstadt, Germany) were used to plot the standard curve.

### Fatty acid profile

The fat extracts were methylated in accordance with ISO 15884 (2002)*.* The fatty acid profile was determined via gas chromatography using a flame ionization detector (Agilent Technologies, Santa Clara, USA). Experimental conditions were as follows: CP-Sil 88 capillary column (100 m, 0.25 mm, 0.20 μm) (Agilent Technologies J&W), temperature gradient: 60 °C (1 min) to 180 °C at Dt = 5 °C/min, injector and detector temperatures: 225 and 250 °C respectively, carrier gas—helium, carrier gas flow rate: 0.8 mL min^−1^, split: 1:100, sample volume: 1 μL, and liner—0.4 mm. BCR-164 anhydrous milk fat (LGC Standards, Poland) served as the reference material.

### Statistical analysis

Two experiments were performed. The obtained results were subjected to a 2-way analysis of variance with replications. Differences between all treatment means were compared at the 5% level of significance using Tuckey`s post-hoc test. Results were presented as a mean values ± standard variation. Data were processed statistically in Microsoft Excel (Microsoft Office 365).

## Results and discussion

The MBMP and MSMP samples for yoghurt production had a dry matter content of 13% ± 0.1% and milk fat content of 1.5% ± 0.06%.

### Rates of acidification

**Fig. 1:**
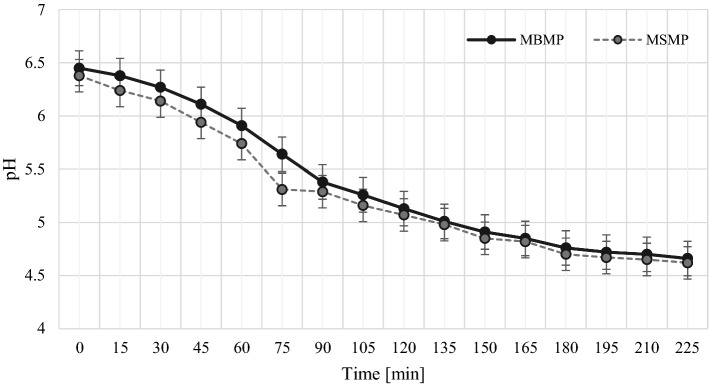
Acidification curves of milk with added buttermilk powder (MBMP) and milk with added skimmed milk powder (MSMP), mean values ± standard deviation (*n* = 2)

The rates of acidification did not differ significantly between the milk with buttermilk powder (MBMP) and the milk with skimmed milk powder (MSMP) added for standardization of dry matter content of the milk (Fig. 1). After inoculation with the starter culture (*Lactobacillus delbruecki ssp. bulgaricus* and *Streptococcus thermophilus)*, the time of acidification was 225 min and was the same for the two powder additives. In contrast, the changes in pH during the process indicated that the acidification rate was slightly higher for MSMP than for MBMP. This negligible difference was evident at minute 75 of acidification at pH 5.3. However, further rates of pH decrease (< 5.3) across acidification time were almost identical for both types of milk (Fig. 1).

Romeih et al. (2014) conducted a similar study on the use of buttermilk powder additives in yoghurt production, which differed from the present study due to the use of skimmed buffalo milk. Their study did show that milk supplemented with buttermilk powder was quicker to acidify than the milk supplemented with skimmed milk powder. The authors attributed the faster acidification rates of MBMP to the higher content of peptides or amino acids of lower molecular weight (used by *Streptococcus thermophilus*) in buttermilk in comparison with skimmed milk. The total time to acidification was recorded at 225 min (Fig. 1) and did not change between the types of powder used to standardize the milk, which corroborated the results of Le et al. (2011) and Romeih et al. (2014). According to Zhao et al. (2020) addition of buttermilk decreased the pH at the gelation point, shortened the gelation time and the fermentation period.

During the analysis of the results, 2-way ANOVA showed a statistically significant influence of the type of product, storage time and the interaction on titratable acidity and lactic acid content in the tested samples (*P* ≤ 0.05, Table 1). The acidity (expressed in pH), titratable acidity, and lactic acid content of both yoghurts were on similar level after 1 day of storage (Table 1). Prolonged storage led to a decrease in the yoghurt pH, but caused no differences between the yoghurt variants across the analogous chilled storage time (*P* > 0.05). In contrast, differences between the yoghurt variants in terms of titratable acidity and lactic acid content did emerge after 7- and 21-day storage (*P* ≤ 0.05). The titratable acidity and lactic acid content values were higher for YBMP compared with YSMP. The differences in lactic acid content in the analyzed samples were particularly evident after 7 days of storage, though the gap lessened after 21 days of storage (Table 1). Similar studies were conducted by Bonczar and Wszołek (2002), demonstrating a decrease in pH and an increase in titratable acidity caused by the activity of lactic fermentation bacteria, which continue the conversion of lactose to lactic acid during storage.

**Table 1:** pH, titratable acidity and lactic acid content of yoghurt during storage

Days of storage at 4 °C		pH		Titratable acidity [°SH]		Lactic acid [%]	
		YBMP	YSMP	YBMP	YSMP	YBMP	YSMP
1		4.45 ± 0.01^a^	4.46 ± 0.03^a^	45.1 ± 0.1^a^	45.0 ± 0.3^a^	1.015 ± 0.002^a^	1.013 ± 0.002^a^
7		4.37 ± 0.01^b^	4.37 ± 0.01^b^	51.4 ± 0.6^b^	47.6 ± 0.6^b^	1.157 ± 0.009^b^	1.071 ± 0.009^b^
21		4.26 ± 0.01^c^	4.27 ± 0.01^c^	52.4 ± 0.6^c^	51.3 ± 0.1^c^	1.179 ± 0.009^c^	1.154 ± 0.002^c^
Significance (*P* value)	Product	not significant		0.000		0.000	
	Storage	0.000		0.000		0.000	
	Interaction	not significant		0.002		0.002	

Values are means ± standard deviation (*n* = 2); YBMP—yoghurt with added buttermilk powder; YSMP—yoghurt with added skimmed milk powder

Mean values in columns (for the same product) with different superscripts are significantly different (*P* ≤ 0.05); experimental factor: days of storage

The increase in lactic acid content was much slower at the chilled storage temperature (about 4 °C) (Table 1) than at the typical ripening temperature for yoghurt starter cultures, as indicated by changes in pH values (Fig. 1).

### Yoghurt syneresis

**Fig. 2:**
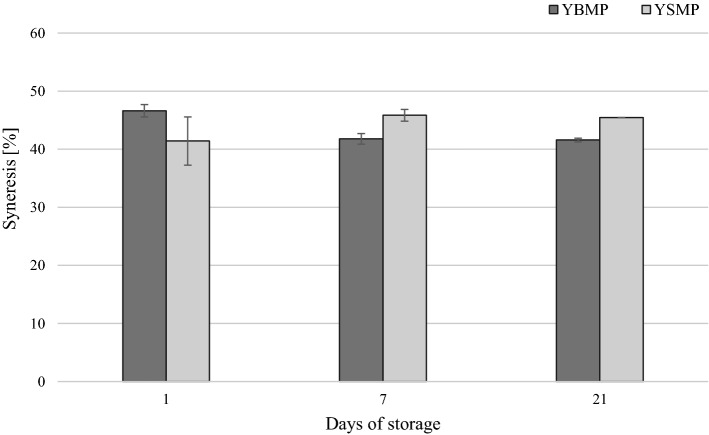
Syneresis of yoghurt during storage. YBMP—yoghurt with added buttermilk powder; YSMP—yoghurt with added skimmed milk powder. Mean values ± standard deviation (*n* = 2), differences not significant

Figure 2 shows the effect of the type of powder used in the yoghurt production mixture on the syneresis of the final product throughout storage.

A change of approximately 5% was detected for YSMP (increase) and YBMP (decrease) syneresis during storage. Yoghurt syneresis often occurs during storage and constitutes a serious defect. As such, reducing whey separation represents an improvement for the consumer. The results are corroborated by studies of Le et al. (2011), Romeih et al. (2014), Trachoo and Mistry (1998) and Zhao et al. (2020) who demonstrated lower values of syneresis for the yoghurt with buttermilk, as buttermilk constituents provide better water holding capacity.

Given that the dry matter content of both yoghurts was the same, the reduced (not significantly) syneresis of YBMP compared with YSMP (Fig. 2) was explained by the increased hydration capacity of its constituents, in particular MFGM proteins and phospholipids. The amphiphilic properties of phospholipids and other constituents of milk fat globule membranes may translate into a higher capacity for water retention.

### Phospholipids in yoghurt

**Fig. 3:**
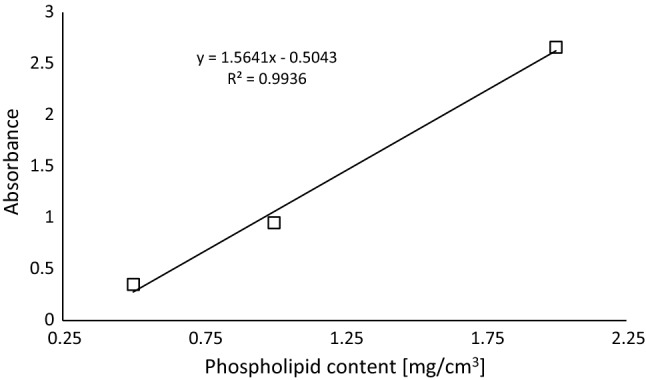
Absorbance of the standard phospholipid solution

The standard curve was plotted based on standard solutions with defined phospholipids concentrations (Fig. 3). The high coefficient of determination for the linear relationship between absorbance and phospholipids concentration points to the validity of the employed method.

During the analysis of the results, 2-way ANOVA showed a statistically significant influence of the type of product and storage time on phospholipid content in the tested samples (*P* ≤ 0.05, Table 2). The present study demonstrated lower (*P* ≤ 0.05) phospholipids contents in the milk fat from the yoghurt supplemented with buttermilk powder, as compared with the yoghurt with skimmed milk powder. The proportion of phospholipids in milk fat in the fermented products decreased during storage (*P* ≤ 0.05). The variation in phospholipids content between the two types of powder was particularly evident in yoghurt after 21 days of storage. Throughout the 21-day storage, the phospholipids content decreased faster in YBMP, maximally by ca. 50%, whereas the decrease in YSMP amounted to about 20% compared to the similar samples stored for 1 day. After storage, the phospholipids content in YSMP was 50% higher than in YBMP (*P* ≤ 0.05).

**Table 2:** Phospholipid content in yoghurt

Days of storage at 4 °C	Type of yoghurt	Phospholipid content [mg/g of fat]
1	YBMP	99.6 ± 2.9^a^
	YSMP	127.4 ± 0.4^a^
7	YBMP	98.0 ± 4.2^a^
	YSMP	118.8 ± 2.5^a^
21	YBMP	67.6 ± 2.6^b^
	YSMP	104.8 ± 7.9^b^
Significance (*P* value)	Product	0.000
	Storage	0.000
	Interaction	not significant

Values are means ± standard deviation (*n* = 2); YBMP—yoghurt with added buttermilk powder; YSMP—yoghurt with added skimmed milk powder

Mean values in columns (for the same product) with different superscripts are significantly different (*P* ≤ 0.05); experimental factor: days of storage

The high phospholipids content in YBMP results from the fat globule membranes being released into the buttermilk during the churning process, whereas in the case of YSMP, it is caused by the high content of very small fat globules, as well as the extensive interface surface of the skimmed milk. In both cases, the addition of the powder products resulted in a high proportion of phospholipids in milk fat (Table 2).

The presence of small fat globules results in a higher content of polar lipids in the product compared to the large fat globules (Lopez 2011). Conway et al. (2014), Mac Gibbon (2006), and Rombaut and Dewettinck (2006) reported that the content of polar lipids was high in both buttermilk and skimmed milk relative to total fat. Although the phospholipids content of buttermilk is 10 times higher than that of skimmed milk, the proportion of phospholipids to total fat is similar in both products (Conway et al. 2014; Mac Gibbon 2006).

The fact that the phospholipids content changes less radically in YSMP compared to YBMP during storage can most likely be explained by the phospholipids from buttermilk-powder MFGM fragments being more susceptible to conversion than the phospholipids present on the surface of fat globules in skimmed milk powder. Further research is, however, needed to explain why those specific changes in the phospholipids content occur in the dry matter of yoghurt fortified with buttermilk powder.

### Fatty acid profile

The fatty acid profile of yoghurts did not vary depending on the composition of the production mixture (Table 3).

**Table 3:** Fatty acid profile of yoghurt

Fatty acid g 100 g^−1^ total fatty acids	YBMP	YSMP
C 4:0	2.82 ± 0.06	2.75 ± 0.09
C 6:0	1.96 ± 0.10	1.92 ± 0.05
C 8:0	1.25 ± 0.02	1.23 ± 0.05
C 10:0	3.03 ± 0.06	2.99 ± 0.07
C 12:0	3.73 ± 0.18	3.69 ± 0.12
C 14:0	12.89 ± 0.27	12.79 ± 0.18
C 14:1	1.06 ± 0.02	1.09 ± 0.09
C 15:0	1.13 ± 0.09	1.16 ± 0.08
C 16:0	36.48 ± 0.28	36.45 ± 0.42
C 16:1	1.84 ± 0.09	1.89 ± 0.06
C 18:0	11.02 ± 0.18	11.02 ± 0.16
C 18:1 t9	1.43 ± 0.13	1.39 ± 0.10
C18:1 c9	20.20 ± 0.93	20.42 ± 0.89
C18:2 c6	1.17 ± 0.07	1.22 ± 0.08

Values are means ± standard deviation (*n* = 2); YBMP—yoghurt with added buttermilk powder; YSMP—yoghurt with added skimmed milk powder. Differences not significant

The literature data (Fauquant et al. 2005; Jhanwar and Ward 2014; Sanchez-Juanes et al. 2009) indicates that the composition of fatty acids can differ depending on their source of origin. The fatty acid profile of phospholipids from buttermilk fat globule membranes differs from that of fat extracted from whole milk. Such phospholipids not only have lower levels of stearic acid and higher levels of oleic and linoleic acids, but also contain very long chain fatty acids (VLCFA), larger than C20 (Sanchez-Juanes et al. 2009), and more unsaturated fatty acids—in particular PUFAs (Polyunsaturated Fatty Acids), whose levels are 6 times higher on the membrane than in the inner core of the globule (Fauquant et al. 2005; Jhanwar and Ward 2014).

In the present study, the use of buttermilk powder in yoghurt production caused no changes in the respective fatty acid profile (Table 3). This is most likely due to the milk fat content being higher relative to the membrane lipids, as well as to the similar phospholipids content in yoghurts with added buttermilk powder and yoghurts with added skimmed milk powder (Table 2).

## Conclusion

The boosted wholesomeness of food is mostly determined by the presence of bioactive substances. In this regard, naturally fermented milk beverages produced using sweet buttermilk can be considered beneficial to human health. The yoghurts with added buttermilk contained less phospholipids when compared with the yoghurts supplemented with milk powder. No differences were found in fatty acid profiles of the two yoghurts with different powders used as additives. The use of buttermilk powder in yoghurt production did not disrupt the milk fermentation process. It did, however, contribute to the increased lactic acid content and a higher water-holding capacity of the stored yoghurt. The reduction in yoghurt syneresis after buttermilk powder addition can be considered as an improvement in product quality, particularly from the consumers’ perspective. Thus, we can conclude that buttermilk can be used as an additive to produce a novel yoghurt type with functional features.

## Data Availability

All data generated or analyzed during this study are included in this published article.

## Abbreviations

MFGM: Milk fat globule membrane
BMP: Buttermilk powder
SMP: Skimmed milk powder
MBMP: Milk with buttermilk powder
MSMP: Milk with skimmed milk powder
YBMP: Yoghurt made from milk with buttermilk powder
YSMP: Yoghurt made from milk with skimmed milk powder
